# An Emerging Role for the Delta Opioid Receptor in the Regulation of Mu Opioid Receptor Function

**DOI:** 10.1100/tsw.2007.219

**Published:** 2007-11-02

**Authors:** Raphael Rozenfeld, Noura S. Abul-Husn, Ivone Gomez, Lakshmi A. Devi

**Affiliations:** Department of Pharmacology and Systems Therapeutics, Mount Sinai School of Medicine, New York, NY 10029, USA

**Keywords:** Opiate Tolerance, G protein-couple receptor heterodimerization, enkephalin, addiction and drug abuse, beta-arrestin mediated signaling

## Abstract

Morphine and related opiates are commonly used in the clinical management of various types of pain. However, the antinociceptive properties of morphine are often overshadowed by the development of tolerance and dependence following its chronic use. The mechanisms underlying opiate tolerance are not fully understood, but appear to involve numerous and complex physiological adaptations. Recently, a role for the heterodimerization of mu and delta opioid receptors in the development of morphine tolerance has been proposed. This novel mechanism could help us to understand several observations, such as the critical role of delta opioid receptor regulation, the impact of delta opioid receptor binding site occupancy, and the participation of beta-arrestin2, in the development of morphine tolerance.

## INTRODUCTION

Opioid receptors belong to the superfamily of G protein coupled receptors (GPCR) that are characterized by a conserved structure consisting of an extracellular N-terminal domain, seven transmembrane domains, and a C-terminal intracellular tail. These receptors convert extracellular signals into an intracellular response through the activation of intracellular coupled G proteins, which, in turn, activate a number of intracellular molecules. The opioid receptor subfamily comprises mu, delta, and kappa opioid receptors (MOR, DOR, and KOR; also known as MOP, DOP, and KOP receptors), which have a wide distribution in the brain and the spinal cord[1,2]. These receptors are activated by the endogenous peptides Met- and Leu-enkephalin, beta-endorphin and dynorphins, and by exogenous opiates such as morphine.

The opioid system has been extensively studied, first using pharmacological tools, such as morphine and its derivatives, and later using agonists or antagonists with MOR, DOR, or KOR selectivity. Opioid receptors, on activation by exogenous or endogenous ligands, modulate nociception, reward, and responses to stress. Stimulation of these receptors also influences respiration, gastrointestinal motility, and endocrine and immune physiology. Cloning of the opioid receptors[3,4,5,6,7] and genetic manipulation of the opioid system (such as the development of knockout mice) has revealed specific roles for the different receptor types. While it is clear that morphine-induced analgesia is mediated by MOR activation (since this phenomenon is lost by MOR gene deletion[8]), the role of DOR in analgesia remains unclear since, in some cases, analgesia induced by DOR ligands has been shown to be mediated by MOR[9]. One mechanism for a role for DOR in modulating analgesia is via receptor heterodimerization. MOR-DOR heterodimerization has been shown to play an important role in the loss of opiate analgesic potential as well as in the development of tolerance[10,11]. In this review, we analyze the different mechanisms thought to underlie the development of opiate tolerance in light of the recent findings with the regulation of DOR trafficking and its consequences on MOR function (summarized in Table 1).

**TABLE 1**
**Summary of the Different Mechanisms Thought to Underlie the Development of Opiate Tolerance, with an Emphasis on MOR-DOR Interaction**

| Mechanism | Paradigm Used | Ref. |
|---|---|---|
| *Adaptation at the level of neuronal networks* | | |
| Excitatory amino acid receptor activation | NMDA receptor antagonist | [17,18,19] |
| Increased release of substance P and CGRP | CGRP receptor antagonist | [25,27] |
| | NK1 receptor antagonist | [26] |
| *Adaptation at the level of the opioid receptors* | | |
| Increased DOR plasma membrane insertion | CGRP gene deletion | [11] |
| MOR desensitization | Beta-arrestin2 gene deletion | [34,35] |
| *MOR-DOR interaction* | | |
| MOR-DOR heterodimer formation | DOR gene deletion | [48,49] |
| | CGRP gene deletion | [11] |
| MOR-DOR specific signaling network | Beta-arrestin2 gene deletion | [34,35] |
| | DOR-selective antagonist | [10,50,58,63] |

## Opiate Tolerance

Morphine and related opiates are commonly used in the clinical management of various types of pain, including cancer pain. The use of opiate drugs in the treatment of chronic pain, however, is limited by a rapid development of opiate tolerance and physical dependence. Opiate tolerance is defined as a gradual loss of drug potency or efficacy, and reduced duration of action. When tolerance occurs, dose escalation becomes necessary to maintain the same analgesic effect, which results in an increased likelihood of adverse side effects, such as nausea, constipation, and respiratory depression.

Tolerance and cross-tolerance to the analgesic effects of opiates have been clearly shown to develop following chronic opiate treatment[12]. In one study, patients receiving the opiate agonist methadone developed pronounced tolerance to its effects as well as cross-tolerance to morphine[13]. Several clinical studies have shown that opiates, particularly morphine, lack analgesic efficacy in neuropathic pain[14] and this has been attributed to the rapid development of tolerance[15]. Opiate tolerance can also be produced experimentally by chronic systemic or regional administration of morphine or other opiate agonists. However, the mechanisms underlying the genesis of an opiate-tolerant state are thus far poorly understood. The diverse theories regarding opiate analgesic tolerance suggest that this complex phenomenon may involve adaptations at several levels. These are discussed below.

## Adaptations at the Level of Neural Networks

Network-based adaptations contributing to the development of opiate tolerance involve the altered activity of excitatory amino acids, neuropeptides, and their intracellular messengers[16]. During pain transmission, excitatory amino acids (such as L-glutamate/L-aspartate) and neuropeptides (such as CGRP [calcitonin-gene related peptide] and substance P) are released from primary afferent nerve terminals to act on their postsynaptic receptors – the *N*-methyl-D-aspartate (NMDA) receptor, CGRP receptor, and neurokinin 1 (NK1) receptor, respectively. This causes the production of second messengers, such as prostaglandins and nitric oxide in the projection neuron, which feed back to the primary afferent neuron to enhance presynaptic neurotransmitter release. Opiates decrease pain transmission presynaptically by reducing neurotransmitter release and postsynaptically by hyperpolarizing the projection neuron. Thus, adaptations in presynaptic excitatory amino acids or neuropeptide release, in their receptor activity or in intracellular messenger activity, may reduce opiate action and contribute to the development of opiate tolerance.

Indeed, excitatory amino acid release and NMDA receptor activation have been shown to play an important role in opiate tolerance. Pharmacological blockade of the NMDA receptor with antagonists such as MK-801 effectively inhibits the development of spinal morphine tolerance[17,18,19], suggesting that chronic morphine treatment induces a compensatory increase in spinal NMDA receptor activity that opposes morphine action. In addition, opiates have been shown to modulate the presynaptic release of CGRP and substance P, which can be colocalized with each other[20] and/or with L-glutamate[21]. Like L-glutamate, CGRP and substance P are physiological antagonists of opiate activity and are thought to be involved in the genesis of opiate tolerance. Studies have shown that chronic morphine treatment causes increased CGRP immunoreactivity (CGRP-IR) in the superficial layers of the dorsal horn[22,23] as well as in an *in vitro* model of cultured adult dorsal root ganglion (DRG) neurons[24]. Coadministration of $CGRP_{8-37}$, a CGRP receptor antagonist, with morphine inhibits the development of morphine tolerance *in vivo*[25] and prevents the increase in CGRP-IR seen *in vitro*[26]. $CGRP_{8-37}$ is also able to partially restore spinal morphine antinociception in animals previously rendered tolerant to its effects[27]. It is clear from these observations that CGRP plays a significant role in both the induction and expression of morphine tolerance. Substance P also appears to modulate spinal morphine tolerance in a similar manner. Chronic morphine administration causes an increase in substance P-IR in the dorsal horn, and the NK1 receptor antagonist, SR 140333, inhibits (and reverses) morphine tolerance[26], suggesting that substance P also contributes to the induction and maintenance of opiate tolerance. Interestingly, it has been shown that DOR is present in substance P–containing vesicles[28,29] and that the modulation of neuropeptide release also affects DOR's cell surface expression. This implies that a common mechanism regulates neuropeptide release and DOR expression at the synaptic membrane, and that membrane insertion of DOR could participate in the modulation of morphine analgesia and tolerance. This is discussed below.

## Adaptation at the Level of the Opioid Receptor

### *Role of Beta-Arrestin2*

Opioid receptor–related adaptations include receptor desensitization and down-regulation (the latter has been well demonstrated *in vitro*, but not as well *in vivo*)[30]. Desensitization is a loss of the opioid agonist's ability to produce a pharmacological endpoint (such as inhibition of cAMP), as has been shown to occur during chronic morphine treatment[31]. Beta-arrestin has been shown to play a role in receptor desensitization. Activated receptors are phosphorylated by GPCR kinases (GRK), leading to the interaction of phosphorylated receptors with beta-arrestin1 and 2. This association contributes to the desensitization of opioid receptors by preventing further coupling to G proteins and promoting receptor endocytosis[32,33].

An intriguing role for beta-arrestin2 in opiate analgesia and tolerance has been uncovered by the generation of beta-arrestin2 knockout mice[34,35]. Mice lacking beta-arrestin2 display enhanced behavioral responses and G protein–mediated signaling in response to morphine, which results in the potentiation and prolongation of the analgesic effects of morphine[35]. In addition, these mice fail to develop antinociceptive tolerance[34]. These results are paradoxical since *in vitro* observations suggest that GRKs and arrestins play a minor role in regulating the effects of morphine in the CNS. For instance, stimulation of MOR by morphine in HEK293 cells does not trigger rapid receptor phosphorylation or internalization[36]. Taken together, these studies indicate that, in contrast to the *in vitro* scenarios, beta-arrestin2 significantly regulates morphine responsiveness *in vivo*.

## ***Role of Opioid Receptors Heterodimerization***

### Regulation of DOR Trafficking

The regulation of DOR expression at the plasma membrane has been extensively studied and appears to combine different complex mechanisms. In recombinant systems, only a fraction of the newly synthesized receptor is ultimately transported to the cell surface[37]. The remaining receptors are retained in the ER, where they are retrotranslocated to the cytosol, deglycosylated, ubiquitinated, and degraded by the proteasome[38]. This mechanism can be regulated by membrane-permeable opioid receptor ligands, which are able to facilitate the transport of newly synthesized receptors out of the ER to the plasma membrane and, thus, decrease the portion of precursors that would otherwise be targeted for degradation[39]. Such compounds that facilitate the proper folding of the receptor and escape from the ER quality control system are called “pharmacological chaperones” (Fig. 1A).

In DRG and spinal cord neurons, while the majority of MOR is transported along the constitutive secretory pathway[29], DOR is often retained in large dense-core vesicles[28] that contain substance P and/or CGRP[32] (Fig. 1B). DOR, when expressed in neurons that do not express substance P, is found in diffuse cytoplasmic compartments[11], reminiscent of the proteasome targeting of DOR observed in HEK293 cells[38]. These studies have led to the hypothesis that in unstimulated axons, DOR is sorted via the regulated secretory pathway into vesicles that contain neurotransmitter peptides and principally retained in these vesicular compartments until release[28]. The exocytic release of transmitters or peptide modulators leads to fusion of these vesicles to synaptic membranes, leading to the cell surface expression of DOR. Recent studies have shown that DOR interacts with substance P and this interaction is responsible for sorting of DOR to the large dense-core vesicles[11] (Fig. 1). Following nociceptive stimulation, substance P is released from the central terminals of sensory neurons and activates its receptors on DRG neurons[40,41]. This also leads to regulated insertion of DOR into the synaptic membrane on nociceptive stimulation (Fig. 1C).

These findings can be analyzed in the light of another set of studies examining membrane insertion of DOR in dorsal horn and DRG neurons[42,43]. Pain-related stimuli, such as injection of complete Freud’s adjuvant or capsaicin, lead to an increased DOR plasma membrane density[43]. Plasma membrane insertion of DOR could also be triggered by nociceptive stimulus-induced $Ca^{2+}$ influx that causes exocytosis of the large dense-core vesicles[44]. Taken together, these data suggest that intracellular DOR is located in large dense-core vesicles and translocated to the plasma membrane on nociceptive stimulus-induced secretion of DOR-containing vesicles.

Prolonged morphine treatment also triggers plasma membrane translocation of DOR[42]. This mechanism appears to involve MOR activation since there is a lack of plasma membrane translocation of DOR after prolonged morphine treatment in animals lacking MOR[45] or on cotreatment with a selective MOR antagonist in wild-type animals[42]. It is well known that acute MOR activation suppresses the release of neurotransmitters by preventing $Ca^{2+}$ influx[46]. It is to be noted that prolonged morphine (and not acute morphine) treatment leads to DOR externalization. Many examples of modulation of MOR response on chronic morphine treatment have been reported and the ability to promote the secretion of CGRP/DOR-containing vesicles could be one of the adaptations of MOR function after chronic morphine exposure. Importantly, increased plasma membrane DOR insertion leads to increased DOR-mediated antinociception[11,42]. This is of particular interest since a recent study using MOR and DOR knockout mice suggests that the antinociceptive properties of DOR agonists are mediated by MOR activation[9]. Taken together, these data suggest that only after DOR externalization would the antinociceptive potential of DOR ligands be mediated by DOR. This also suggests that DOR could be involved in the regulation of other physiological processes.

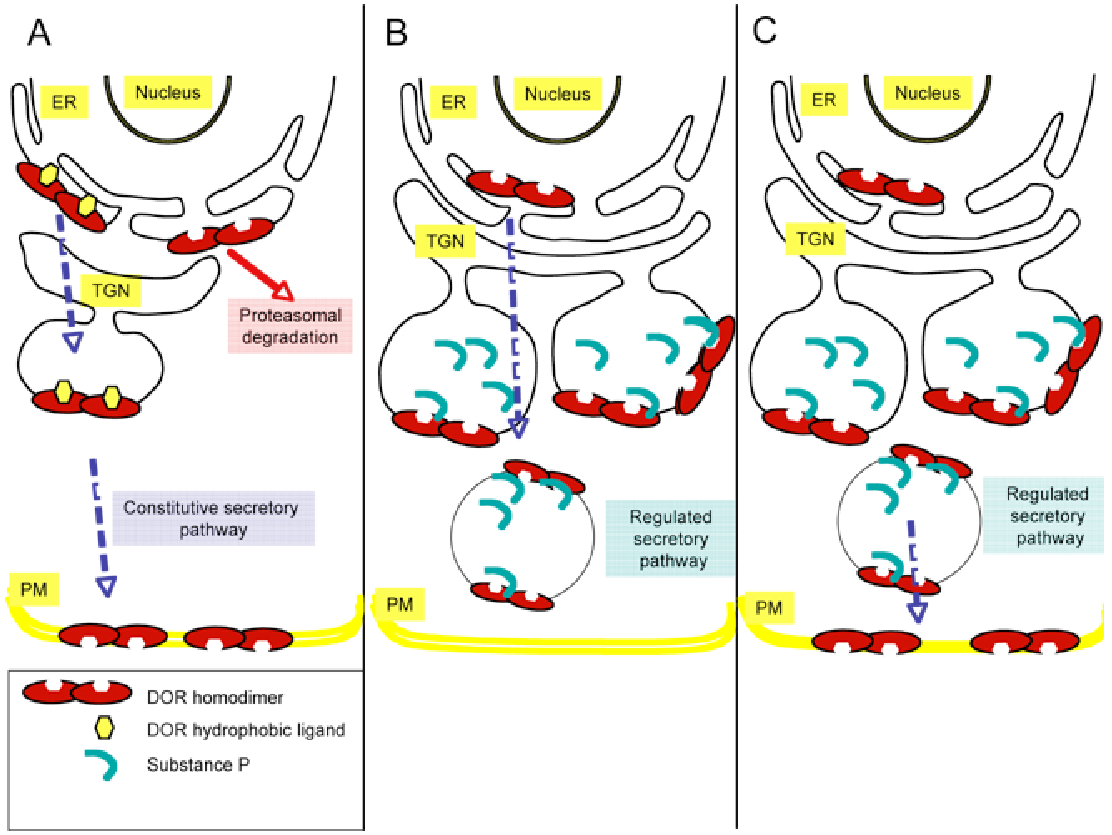

**FIGURE 1.** Trafficking of DOR. (A) In heterologous systems, early after biosynthesis in the ER, the newly synthesized DOR is targeted to proteasomal degradation. Treatment with DOR hydrophobic ligands (also called pharmacological chaperones) leads to DOR maturation and plasma membrane insertion, via the constitutive secretory pathway. (B) In neurons, DOR can also interact with substance P and be sorted to large dense-core vesicles of the regulated secretory pathway. (C) On stimulation, there is neuropeptide release, leading to DOR insertion in the plasma membrane. TGN, trans-Golgi network; PM, plasma membrane.

## MOR-DOR Heterodimerization and Morphine Tolerance

Several lines of evidence indicate that MOR and DOR interact to influence each other's properties[47]. Although morphine acts primarily via MOR[8], DOR is critical for the development of morphine tolerance. Studies with DOR knockout animals have shown that these animals do not develop morphine tolerance[48,49]. Similarly, knockout mice for the preprotachykinin A gene (that leads to reduced cell surface insertion of DOR) do not develop morphine tolerance[11]. Chronic morphine treatment up-regulates DOR[42,45] and this leads to changes in MOR function, such as an altered responsiveness to morphine[11]. Altogether, these results indicate that morphine tolerance requires the presence of functional DOR at the plasma membrane, and that MOR and DOR exhibit functional interaction. This is supported by studies showing that DOR is involved in modulating MOR function at the pharmacological level. For example, mice treated with DOR antagonists exhibit diminished morphine tolerance and dependence[49,50]. The finding that MOR and DOR are coexpressed in the same cells in DRG[51,52,53], and in axonal terminals of the superficial dorsal horn[54,55], further supports the possibility of physical interaction between the two receptors. Mounting evidence for the heterodimerization of MOR and DOR[56,57], and direct interaction of the two receptors in spinal cord membranes, as evidenced by coimmunoprecipitation experiments of native receptors, indicates that MOR and DOR form heterodimers *in vivo*[10]. In cells coexpressing MOR and DOR, DOR-selective antagonists significantly increase the binding of MOR-selective agonists and enhance MOR signaling activity[10,56]. At the physiological level, cotreatment of mice with a DOR-selective antagonist leads to an enhancement of the antinociceptive potential of morphine[10], and combining ultra low doses of DOR antagonists with spinal morphine has recently been shown to augment its acute analgesic effects, inhibiting the induction of tolerance and reversing established tolerance[58]. The formation of distinct signaling complexes via MOR-DOR heterodimerization represents a possible interpretation of these data.

## MOR-DOR Heterodimer as a Distinct Signaling Unit

Heterodimerization of MOR with DOR promotes the formation of novel signaling complexes at the receptors[56,59]. Activation of MOR by selective agonists leads to activation of the mitogen-activated protein kinase (MAPK) cascade and phosphorylation of extracellular signal–regulated kinases 1/2 (ERK 1/2), a mechanism mediated by the G protein-protein kinase C (PKC) pathway[60,61,62]. We have recently found that MOR-DOR heterodimers, however, recruit beta-arrestin2, which leads to a switch in signaling (Fig. 2A). ERK1/2 phosphorylation achieved on stimulation of MOR in complex with DOR displays altered spatial and temporal properties. For example, MOR homodimer–mediated ERK phosphorylation is rapid and transient, and pERK is translocated to the nucleus. In contrast, the MOR-DOR heterodimer–mediated ERK phosphorylation is slower in onset, sustained, and restricted to the cytoplasm (Figs. 2A and B). In addition, this leads to the activation of distinct downstream kinases and transcription factors[59]. Thus, it appears that when present alone, MOR displays optimal coupling and signaling, leading to the “classical” (G protein–mediated) MOR response. MOR, when complexed with DOR, displays impaired coupling and signaling, leading to a “nonclassical” (beta-arrestin–mediated) MOR response. Interestingly, this heterodimer/beta-arrestin–mediated “nonclassical” signaling can be reversed to G protein–mediated “classical” signaling by a DOR-selective antagonist. This (cotreatment with a combination of MOR and DOR ligands) leads to the dissociation of beta-arrestin from the receptors and to a “classical” MOR response (Fig. 2C). The absence of morphine tolerance in animals lacking DOR and or in animals in which DOR insertion in the membrane is impaired is consistent with a requirement of MOR-DOR–mediated signaling in the development of morphine tolerance. Furthermore, the fact that chronic morphine treatment leads to increased DOR surface insertion suggests that morphine tolerance correlates with formation of MOR-DOR heterodimers. Thus, the switch to the “nonclassical” signaling cascade could contribute to the changes in morphine response that underlie tolerance. Coadministration of morphine with a DOR antagonist that would lead to a switch from a “nonclassical” to a “classical” signaling would lead to decrease in the development or reversion of tolerance. This hypothesis is supported by studies showing that animals treated with DOR antagonists exhibit diminished morphine tolerance and enhanced analgesia[10,50,58,63].

There is an interesting parallel between beta-arrestin2 gene deletion and DOR gene deletion in morphine analgesia and tolerance. In both cases, a greater antinociceptive potential of morphine and impaired development of tolerance have been observed. As discussed earlier, the role of DOR in the modulation of morphine effects is likely to occur via heterodimerization with MOR. Beta-arrestin2 is an integral part of MOR-DOR heterodimer–mediated signaling. Therefore, the role of beta-arrestin2 in modulating morphine analgesia and tolerance could be due to its participation in MOR-DOR heterodimer function. This could also explain the observed effect of beta-arrestin2 knockout on MOR function, given that beta-arrestin2 does not significantly interact with MOR alone.

Several factors contributing to the development of opiate tolerance can be examined in light of the novel findings regarding MOR-DOR heterodimers. It is noteworthy that in the study by Abul-Husn et al., not only ultra low doses of DOR antagonists, but also of MOR antagonists, increased morphine effects and impaired the development of tolerance[58]. Heterodimerization has been shown to alter the binding of MOR and DOR ligands to their cognate receptors[56,57]. Thus, it is possible that ultra low doses of MOR or DOR ligands are equally able to bind to the heterodimer and modulate morphine actions by reversing the heterodimer response from the “nonclassical” to the “classical” signaling pathway.

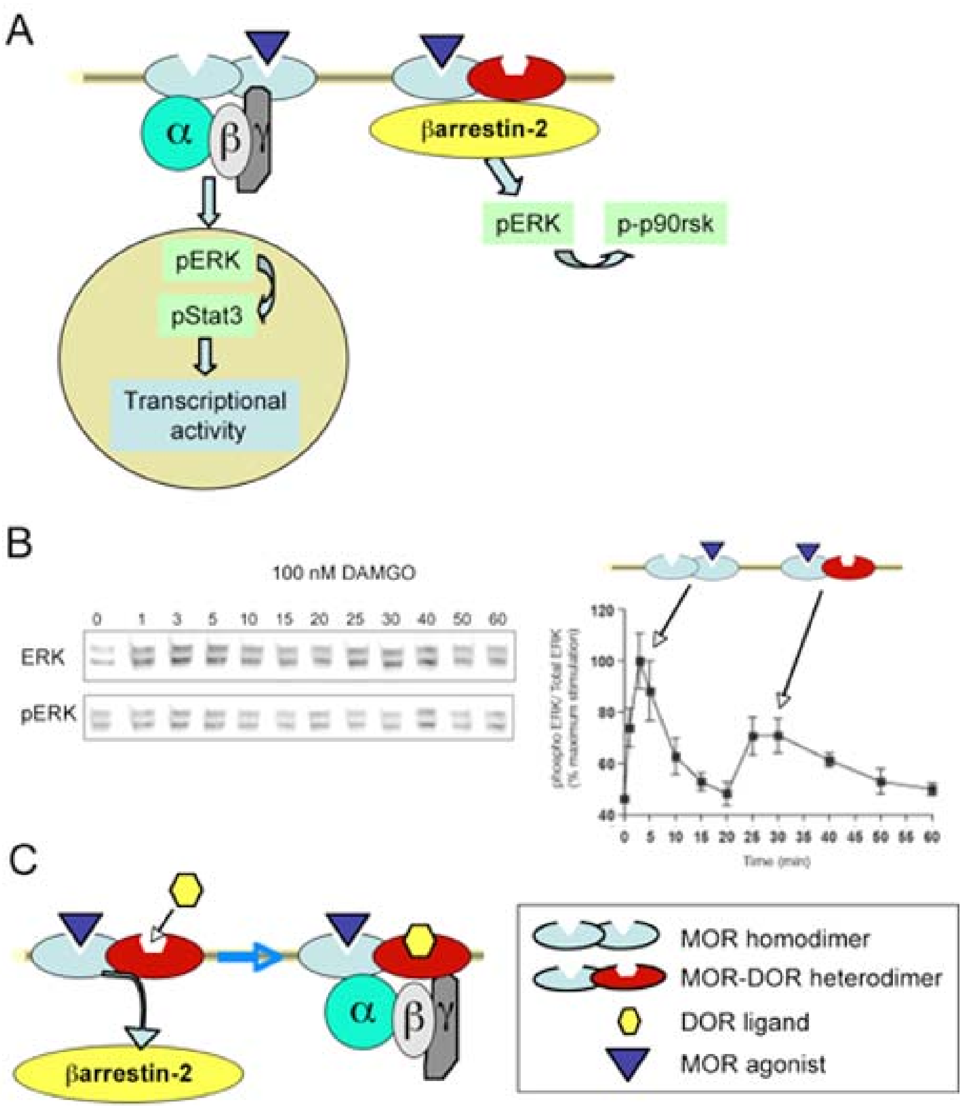

**FIGURE 2.** The "classical" and "nonclassical" MOR signaling pathways. (A) MOR homodimer activation leads to a G protein–mediated ERK1/2 phosphorylation. pERK1/2 goes to the nucleus, where it activates transcription factors. MOR-DOR heterodimer activation leads to a beta-arrestin2–mediated ERK1/2 phosphorylation. pERK is retained in the cytoplsam and activates cytoplasmic substrates, such as p90srk. (B) Illustration of MOR homodimer– and MOR-DOR heterodimer–mediated ERK phosphorylation in primary DRG neurons. Stimulation of the cells with 100 n*M* DAMGO leads to an initial rapid and transient phase of ERK phosphorylation, mediated by MOR homodimers, and a second phase that is slower in onset and more sustained, mediated by MOR-DOR heterodimers. (C) Cotreatment of the heterodimer with a combination of a MOR agonist and a DOR antagonist leads to the dissociation of beta-arrestin2 from the receptor complex, enabling signaling through the "classical" pathway.

## CONCLUSION

Heterodimerization of MOR and DOR represents a relatively new concept in the mechanisms regulating opiate analgesia and tolerance. This concept is very promising because MOR-DOR heterodimers represent a pharmacological target for the development of specific and selective ligands that could achieve analgesic potential without leading to the development of tolerance. Evidence for the critical role of MOR-DOR heterodimerization in morphine tolerance comes from studies showing that chronic morphine leads to an increase of DOR plasma membrane insertion. MOR-DOR heterodimers show less morphine responsiveness than MOR homodimers, but this can be reversed by cotreatment of the heterodimer with a mixture of DOR and MOR ligands. This leads to the dissociation of beta-arrestin2 from the receptor complex, which can then signal through the "classical" G protein–mediated pathway. Further studies are necessary to confirm this model, particularly to assess the increase of the heterodimer/homodimer ratio on chronic morphine treatment, and to correlate this with changes in receptor coupling and signaling.

## ACKNOWLEDGMENTS

This work was supported by NIH grants (DA08863 and DA019521, and NS053751) to LAD.
